# Photovoice and health inequalities among young people in the MENA region: Scoping review

**DOI:** 10.1186/s12939-025-02527-x

**Published:** 2025-06-16

**Authors:** Noorah Aman, Clare Coultas, Jamie Murdoch, Seeromanie Harding

**Affiliations:** 1 Department of Population Health Sciences, School of Life Course & Population Sciences, Faculty of Life Sciences and Medicine, King’s College London, London, UK; 2 Department of Dental Public Health, Faculty of Dentistry, King Abdulaziz University, Jeddah, Saudi Arabia; 3 School of Education, Communication, and Society, Faculty of Social Science & Public Policy, King’s College London, London, UK

**Keywords:** Photovoice, Participatory research, Social determinants of health, Young people

## Abstract

**Background:**

Young people in the MENA region face significant challenges due to socio-political instability, ongoing conflicts, and inequitable social and economic policies. These factors, combined with global threats like climate change and economic instability, hinder the potential of the region's 140 million young people aged 10–24. Addressing these compounded crises is crucial for the future of the region. It is essential to understand the contextual factors shaping young people's health outcomes through their own perspectives. Photovoice, a participatory research method, has shown promise in engaging young people in research.

**Objective:**

This scoping review aims to map the literature on photovoice studies that addressed health and its determinants among young people in the MENA region. It also seeks to highlight the challenges and strengths of employing the photovoice methodology in this context.

**Inclusion criteria:**

The review included literature reporting photovoice projects that addressed young people's health and/or its social determinants, where participants took photos and engaged in discussions based on these images. Studies involving young people aged 10–24 years and focusing on photovoice in the MENA region were considered. Both peer-reviewed journal articles and grey literature with sufficient information addressing the review questions were included.

**Methodology:**

The review followed the JBI Scoping Review Methodology and involved searches of seven English databases, two Arabic databases, and grey literature through Google search.

**Results:**

Eleven studies/projects were included in the analysis. Most of the literature came from non-profit organizations, with few studies from peer-reviewed articles. The included studies focused on socio-economically disadvantaged, vulnerable, and marginalized young people, addressing topics such as environment, social integration, safety, and youth empowerment. The implementation of photovoice varied across studies, and there was limited participation of young people throughout the research process.

**Conclusion:**

The scoping review revealed a scarcity of literature on the use of photovoice among young people to address health inequalities and the factors influencing them in the MENA region. Given the public health value of photovoice as an action-oriented research approach that promotes meaningful participation from young people, further research is needed to leverage this methodology to tackle health inequalities effectively.

**Supplementary Information:**

The online version contains supplementary material available at 10.1186/s12939-025-02527-x.

## Introduction

Young people are experiencing escalating mental health issues worldwide amidst a world of crises and uncertainty, a trend accelerated by the COVID-19 pandemic [1]. This is of significant concern in the Middle East and North Africa (MENA) region, where young people face an even more uncertain context [2, 3]. The MENA region is characterized by enduring conflicts and houses a substantial segment of the global refugee population [4]. Over nine million individuals have sought asylum in the region, including approximately 39% under the care of the United Nations High Commissioner for Refugees (UNHCR) and about 63.33% of the refugees in the region are Palestinian refugees registered with The United Nations Relief and Works Agency (UNRWA) [4]. Beyond global threats like climate change and economic instability, the MENA region’s socio-political instability, ongoing conflicts, and inequitable social and economic policies severely impact young people's ability to reach their full potential [2, 5]. With the future of the region dependent on the development of its 140 million young people aged 10–24 [6], addressing these compounded crises is imperative.

The MENA region exhibits significant diversity in its economies, geographies, infrastructure, and resource endowments [7] (Table 1). It comprises six high-income Gulf Cooperation Countries (GCC), alongside Iraq and Libya as upper-middle-income nations, and nine low-middle-income countries, with Sudan, Syria, and Yemen classified as low-income. Most of these nations display relatively high Human Development Index (HDI) scores, with the GCC countries leading, while Yemen records the lowest HDI score at 0.4. Income inequality is pronounced in nearly half of the region's countries, notably Sudan ranking highest in this regard. Djibouti, Sudan, and Yemen report the highest mortality rates among their peers. Among the twelve countries documenting child poverty, Sudan and Yemen report the highest prevalence rates.

**Table 1:** Socioeconomic context of MENA countries

	**Country**	**Income classification **^**a**^	**Human Development Index **^**b**^	**Gini index **^**c**^	**Mortality rate age 10–19 **^**d**^	**% Children poverty **^**e**^	**% Teenage pregnancies **^**f**^
1	**Bahrain**	High Income	0.888	34.6	2.56		
2	**Kuwait**	High Income	0.847	40.6	2.84		
3	**Oman**	High Income	0.819	40	3.77		
4	**Qatar**	High Income	0.875	34.6	2.56		
5	**Saudi Arabia**	High Income	0.875	34.6	4.90		
6	**United Arab Emirates**	High Income	0.937	26	2.66		
7	**Iraq**	Upper Middle Income	0.673	40.8	6.63	23.20%	14.1
8	**Libya**	Upper Middle Income	0.746	40.1	3.97		
9	**Algeria**	Low Middle Income	0.745	33.5	3.69	6.30%	1.1
10	**Djibouti**	Low Middle Income	0.515	43.7	14.81	22.30%	
11	**Egypt**	Low Middle Income	0.728	35.4	5.52	33.90%	6.7
12	**Iran**	Low Middle Income	0.78	43.2	6.14		5.2
13	**Jordan**	Low Middle Income	0.736	40	4.04	16.10%	5
14	**Lebanon**	Low Middle Income	0.723	35.8	5.27	32.40%	
15	**Morocco**	Low Middle Income	0.698	42.2	2.98	5.10%	7.5
16	**Tunisia**	Low Middle Income	0.732	39.4	4.63	15.20%	0.7
17	**West Bank and Gaza**	Low Middle Income	0.716	44.9	4.77	33.50%	5.9
18	**Sudan**	Low Income	0.516	53.5	12.56	49%	21.5
19	**Syria**	Low Income	0.557	35.3	5.82	8.7	
20	**Yemen**	Low Income	0.424	39.6	10.27	51.50%	16.9

^a^ Classification according to the World Bank

^b^ Human Development Index (HDI) based on UNDP data 2021

^c^ Gini based on WIID (https://www4.wider.unu.edu/). Values on the Gini index represent Year 2020 for all countries

^d^ Mortality rate age 10–19 Unit of measure: Deaths per 1,000 children aged 10 (Year 2022)

^e^ Children living in households with income below the national poverty line (as a % of all children) Time period: 2020

^f^ Early childbearing—Percentage of women aged 20–24 who gave birth before age 18. Source MICS and DHS 2003−2020

A recent UN report on the health and wellbeing of young people in the MENA region showed that non-communicable diseases account for 49% of the total disease burden among girls, followed by mental disorders (22%) and injuries (14%) [6]. Among boys, injuries were the leading cause of poor health (37%), followed by non-communicable diseases (36%) and mental disorders (16%) [6]. While heavy episodic drinking was uncommon among young people in the region, there was a higher rate of consumption of carbonated soft drinks in almost all the countries with available data [6]. Almost half the countries in the region had high prevalence of overweight individuals [6]. The prevalence was higher among males than females, with the highest prevalence in Kuwait (females: 40%, males: 45%) and the lowest in Sudan (females: 16%, males: 8%) [6]. There was limited data on the reproductive health needs of young people, particularly for unmarried individuals [6]. Syria (21.5%), Yemen (16.9%), and Iraq (14.1%) had the highest percentages of teenage pregnancies [6]. Research is needed on the drivers of inequalities in health among young people to identify their needs, priorities, especially in relation to refugees and displaced individuals.

Globally, there is increasing recognition of the importance of young people’s participation in shaping policies and programs that affect their health and well-being [7–9]. The pivotal roles of young people in the movements such as the Arab uprisings, the Black Lives Matter, U.S. gun control protests, and international climate strikes have highlighted their efforts to secure rights related to health, economic opportunities, political expression, and education [10]. Engaging young people in research that directly impacts their lives can provide unique insights not typically seen by adult researchers which may create new opportunities for positive change [11]. Involving young people from the outset can help ensure that research questions are relevant, appropriate tools are selected, and findings are interpreted effectively, thus integrating their perspectives is key in planning research and relevant health programmes. [11, 12]. It is essential, however, to ensure that participation with young people is conducted in a meaningful and ethical manner [10].

Photovoice is a participatory method that has promoted engagement of young people in research [13, 14]. It is a participatory action research method that integrates health promotion principles, critical consciousness education, feminist theory and documentary photography [15]. In the 1990's, Wang and Burris developed photovoice with three primary goals “(1) to enable people to record and reflect their community's strengths and concerns, (2) to promote critical dialogue and knowledge about important issues through large and small group discussion of photographs, and (3) to reach policymakers” [16]. The process involves participants taking photos of their lived realities and experiences with the aim of addressing their health issues and concerns for change purposes. These photos facilitate group discussions and are used to bring attention to policymakers and key stakeholders to influence meaningful improvements to their conditions and to mobilize the desired change [16]. As with other methods of participatory arts such as dance, theatre, photovoice facilitates a reflective inquiry process [16], generating insights that may not be attainable through non-participatory research techniques [16, 17], particularly for topics that may be “difficult to verbalise” [18]. Photovoice can enable young people-generated knowledge and action that is socially relevant with immediate real-world application [11, 16], bridging the gap between research and practice, addressing the "know-do" gap issues in the public health field [15, 16, 19, 20].

There have been two literature reviews of photovoice studies on the health of young people [13, 14], mainly based on studies in United States, China, Canada, East and South Africa, Europe. To our knowledge, there has been no review of Photovoice studies with young people in the MENA region. This scoping review aimed to identify the literature on photovoice studies on the health of young people in the MENA region.

## Review questions

- Q1: What were the health-related issues captured by the studies and how was photovoice used?
- Q2: What were the benefits and challenges of using photovoice as a participatory tool with young people in the MENA region?

## Methods

This review was conducted following the JBI methodology for scoping reviews [21] and reported based on the Preferred Reporting Items for Systematic Reviews and Meta-Analyses extension for Scoping Reviews (PRISMA-ScR) [22]. The protocol for this review can be found in https://osf.io/v38ak.

### Eligibility criteria

This review included literature reporting photovoice projects that addressed young people’s health and/or its social determinants, specifically where participants took the photos and engaged in a discussion based on these images; studies that involved young people aged 10–24 years (based on WHO and UN definitions) [23, 24]; and literature that focused on photovoice in the MENA region based on the UN list. We considered peer-reviewed journal articles, and grey literature for inclusion if it contained sufficient information that addressed the review questions (Table 2).

**Table 2:** Eligibility criteria

Criterion	Inclusion Criteria	Exclusion Criteria
Concept	Photovoice studies that involved participants taking photos of the issues and engaging in discussions based on these photos Studies that focused on young people’s health and/or its social determinants	Studies that reported the use of photovoice without mentioning sufficient details how it was used
Participants	Young people aged 10–24	Studies that included a mix of young people with children and/or an older population and did not separate out the data for the different age groups
Context	Studies conducted in the following countries: Algeria, Bahrain, Djibouti, Egypt, Iraq, Iran, Jordan, Kuwait, Lebanon, Libya, Mauritania, Morocco, Oman, Palestine, Qatar, Saudi Arabia, Sudan, Syria, Tunisia, the United Arab Emirates, and Yemen	Studies conducted with young people who were living outside of the MENA region
Types of sources	Peer-reviewed journal articles Dissertation and other grey literature (if it had sufficient information that addressed at least one of the review questions)	Methodological papers, editorials, commentaries and book chapters with no references to MENA cases studies

### Information sources

We searched for published and grey literature in Arabic and English bibliographic databases and in other relevant sources. We searched 7 English bibliographic databases: *MEDLINE*, *Embase*, *APA PsychInfo, Global Health, CINAHL, Scopus* and *Web of Science*. For the other English sources of information, we searched The Eastern Mediterranean Health Journal (EMHJ) and PhotoVoice.org. For the Arabic search, we searched the *Al Mandumah* and *EBSCO Arab World Research* bibliographic databases. Google was utilized to search for relevant grey literature for both languages.

### Search strategy

We followed 3-steps search strategy. An initial limited search of *MEDLINE* was undertaken to identify relevant articles on the topic. The text words contained in the title and abstract of retrieved papers, and the index terms used to describe the articles were used to develop a full search strategy. We did not include terms for our population in our search strategy to prevent the loss of any relevant studies as there is discrepancy in the literature of how young people are defined. This search strategy was formulated in consultation with an experienced librarian for use in seven English electronic databases and in *Google*. The author NA translated search keywords into Arabic to conduct searches in the Arabic databases. Additionally, certain Arabic translations of Photovoice and its synonyms were derived from the author NA exploration on Google to identify possible translations for Photovoice in Arabic.

The search strategy, including all identified keywords and index terms, were adapted for each included information source. The Ovid search engine platform was used to search for relevant publications in four databases (*EMBASE, MEDLINE, APA PsychInfo, and Global Health*), where each search was run independently. The other five databases were each used by their respective platforms. In addition, the reference list of all photovoice studies, photovoice reviews or reviews that included photovoice among other participatory approaches were manually inspected to identify further relevant studies.

A validated search filter for Arabic countries was employed, with modifications made to tailor it to the specific context of this review [25]. Since Palestine and Israel may be used interchangeably, we initially included Israel as a search term results to ensure comprehensive coverage and prevent overlooking eligible studies of photovoice with Palestinian young people. Any Israeli study that focused on the majority Palestinian youth population was included. No limits on date or language were applied during the searches other than Arabic keywords used for Arabic databases and English keywords used for English databases.

For our Google search, we used the advanced search interface (as detailed in the search strategy file included the supplementary materials). We reviewed the results until the pages were populated with studies that did not meet the eligibility criteria. The search was conducted from August 2023 to 30 April 2024. A copy of the full search strategy can be found in the supplementary material.

### Selection of evidence sources

All identified citations were uploaded into the bibliographic software EndNote 20 (Clarivate Analytics, PA, USA) with citations of each language stored in a separate library. NA screened the retrieved abstract and manually removed any duplicates not identified as exact matches by the automatic removal function in EndNote. The full texts of selected citations were thoroughly assessed against the established inclusion criteria by NA. Any conflict or discrepancies were discussed with CC and SH and resolved through consensus. In cases where the full text of selected citations was not readily accessible, efforts were made to contact the study authors/inter loan library services. It should be noted that some details may have not been reported in documents other than journal articles or dissertations due to their different structures, scopes, and lengths; however, they were included because they reported sufficient details to be relevant to this scoping review.

### Data extraction

The review questions were used as a guide to extract and analyse relevant data. A data extraction spreadsheet was created to gather information from the studies that met the inclusion criteria. The data extraction tool was piloted by NA and modified iteratively throughout this process with CC. Extraction of photovoice procedures was guided by Wang and Burris's methodology [15]. To facilitate reporting, we further grouped these data under photovoice training, instruction and equipment, discussion and analysis and dissemination. Additionally, we used Wang and Redwood (2001) [26] minimum best practices of photovoice ethics to extract data on photovoice ethical aspect and consideration.

### Data items

We extracted data on study characteristics such as year of publication, author/s name, study title, document type, country, topic/field of study and health inequalities/inequities addressed, as well as population information such as age, gender, sample size. Additionally, we extracted data on the aim of the photovoice project, photovoice design and procedures, outcomes relating to health and social determinants, benefits and challenges of using photovoice, and ethical considerations.

### Data analysis and synthesis

We used basic qualitative content analysis as recommended by JBI guidance for scoping reviews [27, 28]. Raw data summaries and categories resulting from both inductive and deductive approaches to the extracted data were charted in tables. Descriptive themes were created when necessary to facilitate a narrative summary of relevant information under each review question. Since we included both peer-reviewed and grey literature materials, we grouped the included studies in the review based on their document types. This was done to facilitate a valid narrative summary, as not all documents contributed equally to answering each review question due to their varying levels of detail.

### Quality assessment

The quality of grey literature materials was assessed based on their origin from reputable organisations, their currency, and their relevance and substantive information for our review before inclusion. To evaluate the reputation of these organisations, we considered their history of producing reliable research, their credentials, and whether they have a clear mission, established history, and a verified location or official registration number with the relevant regulatory body. For the peer-reviewed research articles, we used the Joanna Briggs Institute's (JBI) qualitative checklist to assess the methodological quality of the included studies [29]. While photovoice is firmly grounded in the participatory action research (PAR) paradigm—and would ideally be assessed using a PAR-specific checklist—no widely accepted tool for evaluating PAR-based research is known to the authors. We selected the JBI checklist because, although designed for qualitative research more broadly, it emphasizes congruity between a study’s philosophical perspective, methodology, methods, and interpretation. This flexibility makes it suitable for evaluating diverse qualitative approaches, including photovoice. To further tailor our assessment to this method, we also drew on Wang and Burris’s photovoice framework, which outlines key ethical practices [26] and methodological steps specific to the approach [15, 16]. This combined strategy allowed for a more inclusive and context-sensitive evaluation of the included studies.

## Results

### Study inclusion

**Fig. 1:**
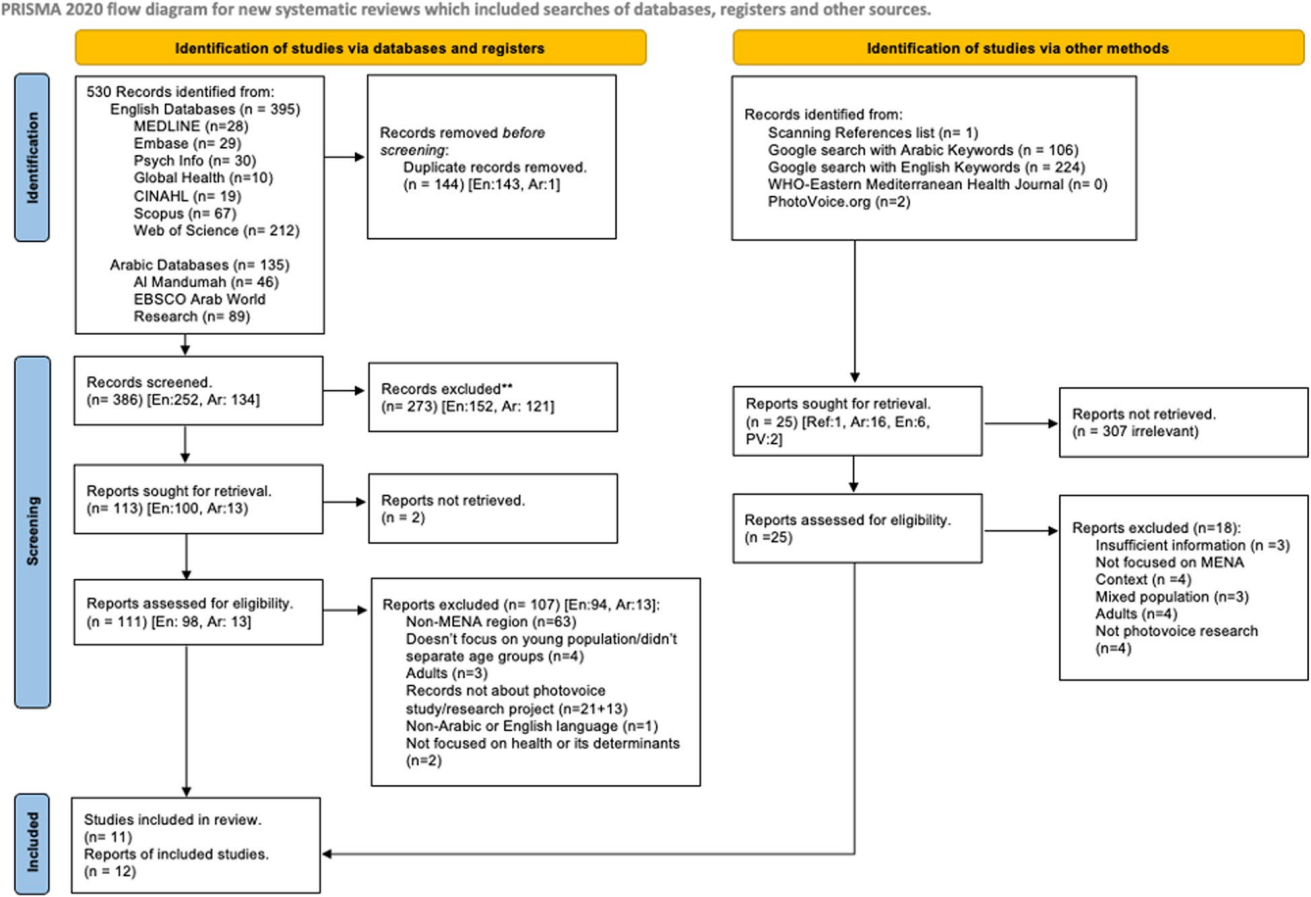
PRISMA Chart for Study Selection

A total of 530 records were identified across 9 databases, with 135 of these from the two Arabic databases. Of those, five studies met all inclusion criteria for this review. An additional 333 records were identified through other search methods. For our Google search, Google filtered out highly similar entries to ensure the most relevant results, yielding 224 English records and 106 Arabic records. Of the 333 records, seven documents met the eligibility criteria. In total, 11 documents satisfied the eligibility criteria for this review (the 12 th is a thesis related to one of the included studies). Figure 1 details the selection process and reasons for exclusion for the databases and other sources searches.

### Characteristics of included studies/reports

The included documents (Table 3 in the Supplementary Material 1), published between 2008 and 2023, were from Egypt (*n* = 3), Jordan (*n* = 1), Lebanon (*n* = 2), Palestine (*n* = 4) and Saudi Arabia (*n* = 2). The review included four peer reviewed journal articles [30–33] (one accompanied by a thesis), three reports from a non-governmental organisation (NGOs) [34–36], a book chapter (which included an example of a photovoice case study) [37], and three news webpages [38–40]. Three documents were in Arabic; one was translated by NA [38], and two had an available English version [34, 40]. Most of these photovoice projects were initiated by international and local non-profit organisations; with one project using photovoice as part of multi-methods study [34]. A few were conducted by academic researchers (*n* = 5) [30–33, 37].

### Quality assessment of included studies

All grey literature documents were of sufficient quality for inclusion, despite variations in the depth of content. Among the peer-reviewed studies (Table 4 in the supplementary document), there was inconsistency in how Photovoice was reported, a pattern that aligns with findings from other Photovoice reviews. Additionally, those studies employed Photovoice differently from the photovoice methodology proposed by Wang and Burris [15, 16]. Almughamisi et al. conducted a concept mapping study using a mixed methods approach that involved various stakeholders. In this study, the Photovoice component was limited to adolescent participants and was used solely to generate statements that informed the concept mapping process. In contrast, the other two studies (both authored by Hayik) integrated Photovoice into English writing courses, involving only students within classroom settings. In terms of methodological congruity—specifically, alignment between Photovoice steps and the methods used for data collection and analysis—none of Hayik’s studies included group discussions or involved youth participants in the analysis process. Additionally, it was not clear whether participants were given opportunities to validate the findings.

Overall, none of the included studies reported all of the minimum ethical practices recommended for photovoice [26] which is consistent with findings from other reviews of photovoice [41]. However, it remains unclear whether these omissions reflect the actual practices used. Only two projects demonstrated a more participatory approach by including group discussions and participatory analysis: the Naba’a study (from the grey literature) and Almughamisi et al.’s study (from the peer-reviewed literature). Despite variations in methodological detail and alignment with Wang’s original Photovoice framework (see Table 4 and 5 in the Supplementary Material 2), we adopted an inclusive perspective. Studies that adapted Photovoice for specific contexts—whether conceptually or methodologically—still provided valuable insights and enriched our understanding of the review topic.

## Review findings

### A) Health-related issues

Overall, one project focused on the prevention of health risk factors [30], while the remaining projects addressed various issues related to social determinants of health [31–40]. Among those peer-reviewed papers, one study addressed the prevention of childhood obesity in Saudi Arabia through a concept mapping approach to identify feasible interventions from the perspectives of adult and adolescent stakeholders. Both stakeholder groups agreed on improving access to healthy foods in the canteen [30]. The other two studies addressed issues faced by minority Palestinian adolescents in Israel. The first study framed the issues as environmental concerns, highlighting problems such as garbage disposal, environmental pollution, problematic use of public space, and deforestation, focusing on how these environmental factors negatively impact the daily lives and well-being of Palestinian adolescents [31]. The second study framed the issues as broader community problems, identifying issues like littering, poor infrastructure, violence, lack of public manners, unhealthy eating habits, child smoking, and social acceptability, focusing on how these community-related problems affect the social and psychological health of Palestinian adolescents [32].

Among the grey literature, three projects focused on supporting factors for young people's health, well-being, and development. One study focused on leadership empowerment to enhance life opportunities for youth in Lebanon [39]. Another was an online project that promoted entrepreneurship to improve work opportunities for Egyptians and other Arab [40]. The third project addressed the challenges of Egyptian orphans transitioning to independence after leaving home care [38].

Five projects addressed various challenges faced by young people. Two focused on refugees; one on sexual violence among displaced Syrian refugees in Jordan [36] and the other on integration of Arab refugees in Lebanon [34]. Both projects, initiated by non-profit organisations, aimed to listen to adolescent experiences [34, 36] and make recommendations for improvement [34]. One study explored safety of Egyptian youth during a time of sociopolitical conflict, highlighting how figures of social order, such as police presence, represent safety symbols for youth [37]. In one study that incorporated photovoice as part of the English writing course syllabus, Palestinian minority students highlighted linguistic landscape issues in their surrounding such as the absence of Arabic from many signs, the abundance of mistakes in spelling Arabic words and the Hebraization of many Arabic place names, and how these issues affected them [33]. Additionally, PhotoVoice, a nonprofit organisation used photovoice with Palestinian and Israeli adolescents to promote mutual understanding within the Palestinian-Israeli conflict [35].

### B) How photovoice was used

Six out of eleven studies (Table 5 in the supplementary documents) provided a rationale for using photovoice [30–34, 37]. From these 6 studies, reasons related to meaningfully engaging marginalised groups to articulate ‘authentic’ experiences [33]; stimulate community interest [34], raise awareness of challenges within communities [31, 33, 37], and as a tool for ‘empowering’ marginalised groups to effect change [31–34].

Three studies used photovoice as participatory research [31, 34, 37], with one using photovoice as part of a multi-method study [34]. One study used photovoice as participatory data collection to enhance adolescents’ involvement in a concept mapping study involving different stakeholders [30]. Additionally, two studies incorporated photovoice projects into the English as a foreign language teaching syllabus, which focused more on critical literacy pedagogy and the participatory documentary elements of photovoice [32, 33].

All of the included projects involved young people in the photo-taking phase. Of the 11 projects, three reported conducting group discussions and included participants in the analysis phase [30, 34, 36]. Another three did not conduct group discussions but instead used an individual exercise in which participants wrote personal narratives about their photos, and the analysis was conducted by the researchers [31–33]. Among the projects that did not involve young people in the analysis phase, the reasons included time and budget constraints (*n* = 1) [37] or differing objectives, such as a focus on education (*n* = 3) [31–33].

Some projects stated resulting in increased awareness and consciousness-raising of the issues they addressed (*n* = 3) and shared their findings locally (*n* = 4) [32–34] or internationally (*n* = 3) [35, 36, 42] towards raising social or global awareness. Others went beyond consciousness-raising, resulting in recommendations for necessary actions (*n* = 1) [34], co-creation of an action plan (*n* = 1) [37], development of a program curricula (*n* = 1) [36], personal and social change of the immediate environment (*n* = 2) [31, 37], discussion with policy makers or influential figures (*n* = 3) [31, 38, 39], dialogue between two conflicted parties (*n* = 1) [35], youth-led projects (*n* = 1) [37] and capacity building (*n* = 1) [39]. The study that used photovoice to generate statements aimed at maximising adolescent participation in the concept mapping study led to developing an intervention in school canteens [30].

### C) Photovoice benefits

*Enhanced communication and expression (n* = *4)* Enhanced communication and expression were seen as a benefit of photovoice across 4 studies [30, 34–36]. One study reported that photovoice enabled students to generate 43 statements from the 39 photos, which enhanced their concept mapping process [30]. Another study highlighted the role of participant photos in facilitating important dialogue between conflicted Palestinian and Israeli groups, providing a platform for adolescents to express their perspectives [35]. Two quotes from different projects illustrate how participants used photovoice to deeply convey their emotions and personal experiences: “This project helped me to deliver my point of view through photography in a better way, because I now look deeper into the lens. For me, I prefer taking photos that expresses how I feel, even if it makes others angry” [36]. *“*The exhibition provides us the chance to display the suffering of the children and youth and the difficult life that hinders integration and pushes to emigrate.” [34].

*Boosted self-confidence (n* = *2)* Two studies noted, based on analysis of students’ reflections, that students felt the photovoice process boosted their self-confidence especially when presenting their work in front of an audience [31, 32]. One student reflected on this: “Presenting my photo and accompanying problem in English to the audience increased my confidence. I was a bit worried to make mistakes since English was my third language, so I practiced a lot at home till I memorized my text. The positive feedback I received from my classmates and professors was very encouraging and satisfying.” [32].

*Raised awareness of the investigated issues (n* = *3)* Three studies noted, based on analysis of students’ reflections, that the photovoice process helped to raise their awareness of things that was previously unseen to them, it was an eye-opening experience for them [31–33]. One student wrote: “I used to hear that the environment was dirty, but I didn’t expect the situation to be so bad. After listening to my classmates’ presentations, I became very angry! I became more conscious of the negative consequences of these problems” [31]. Students also reported that the raised awareness of critical issues through photovoice prompted proactive problem-solving and critical examination of issues: "I believe this project benefited me greatly. It has increased my awareness concerning problematic issues. Now that I went through this activity, I examine things more critically, with an open eye" [32]. A second student noted: "When I was done with the photovoice project, I was astonished by the results. This was my first experience to really look into conflicting issues and try to find solutions that would bring justice to my society" [32]. It was also said to encourage action: "This experience made me start thinking about changing the situation instead of just complaining about it. Merely complaining is not enough. It is very important to suggest solutions and try to implement them." [32].

*Enabled social consciousness raising activities (n* = *2)* Two studies demonstrated how the photovoice process enabled social consciousness-raising activities after the project ended [31, 37]. In Hayik’s (2021) study, the author reported that students became more environmentally active and provided examples evidenced with photos showing students organizing weekly village clean-ups and securing support from the local council for supplies and disposal [31]. The second study provided illustration of how photovoice facilitated such consciousness raising activities: participants formed a group to implement youth-led safety initiatives, addressing the safety concerns they identified in their project [37]. They also organized a dialogue session to prioritize safety modes identified in their collective photo stories in collaboration with the local research team.

*Feeling valued and heard (n* = *3)* Three studies noted that the photovoice process made young people feel valued and heard [32, 34, 37]. In one study that analysed student reflections, students reported that they appreciated the opportunity to express their views. One student wrote: "In the beginning, I was confused. No one has ever asked me to write about things that bothered me..". Similarly, another student in the same study said: "It was a completely different experience for me. Throughout all my schooling years, this was the first time someone asks me to write about what bothered me" [32]. Although the other two studies did not specifically evaluate the benefits of photovoice, they shared participants’ statements that illustrated these feelings. In Malherbe et al.’s [37] study, one participant noted that the project allowed her to “say my opinion freely”, with another stating “our voice has come close to [that of] authorities” [37]. Another project shared one participant’s comment “We were engaged in critical thinking with community leaders. For the first time, it was an opportunity for us to be heard and understood” [34].

### D) Photovoice challenges

Three studies identified challenges associated with the use of photovoice, two of them focused on minority young people in Palestine. In one study [33], the author reported how the heightened awareness resulting from the photovoice process prompted minority young people to confront unseen realities, and perpetuated feelings of marginalization. Another study [32] reported cultural and sociopolitical challenges related to taking photos within their local communities, where there was a fear of legal repercussions. These two studies also highlighted challenges related to the dissemination of photovoice projects among minority young people [32, 33]. They reported fear of negative consequences such as loss of job opportunities, resulting from critiquing authorities or sharing critique with the wider audiences as reasons for young people refusal to disseminate their findings [32, 33]. The third study mentioned how the time and budget constraints in their photovoice project limited the young people involvement in the data analysis, thereby impacting the participatory nature of photovoice [37].

## Discussion

This scoping review investigated how photovoice has been used to address health inequalities among young people in the MENA region, highlighting its benefits and challenges. Most of the included projects focused on addressing social determinants of health such as environment, social integration, safety, and empowerment targeting disadvantaged, vulnerable and marginalised groups. Photovoice implementation varied across studies, reflecting diverse adaptations of the methodology and differing levels of young people involvement throughout the research process. The main benefits were raising awareness and enhanced communication expression of young people, while reporting on challenges and the ethical complexities of conducting this form of research was limited.

Most studies focused on wider determinants of young people’s health and wellbeing, which align with some of the priorities outlined in the recent UN MENA report on young people health and wellbeing [6]. One issue that was addressed was sexual violence among refugees [36]. Adolescents in humanitarian and fragile settings, such as those affected by armed conflicts and natural disasters, face heightened vulnerability due to separation from protective family networks and disrupted access to essential services [43, 44]. Conflict and displacement leave children vulnerable to sexual violence, early marriage, harassment, isolation, and exploitation [43, 44]. This vulnerability often forces them into survival strategies like dropping out of school, early marriage, or transactional sex [43]. In such contexts, the breakdown of governance and health infrastructures drastically reduces access to protective services, leading to alarming rates of preventable mortality and morbidity among young people [43, 45]. Sexual violence has been linked to major effects on physical and mental health, including injuries from rape, HIV, reproductive health problems, social isolation, depression, post-traumatic stress disorder, and self-harm [44, 46]. Addressing the interconnected issues of sexual violence is critical for protecting young people from exploitation and mitigating the long-term impacts of sexual violence in crisis settings.

Three projects focused on protective social determinants for young people's health and wellbeing, including youth empowerment, leadership, work opportunities, and transitions into independence. The Global Accelerated Action for the Health of Adolescents framework identified these social determinants as key domains for adolescent health, social development, and wellbeing [43, 47]. The UN H6 + Technical Working Group on Adolescent Health and Wellbeing agreed that adolescent well-being is achieved when adolescents have the support, confidence, and resources to thrive within secure and healthy relationships, enabling them to reach their full potential [43, 47]. This critical and rapid stage of human development is a formative period, and ensuring the presence of protective determinants is important for youth development to enhance resilience and reduce health risk behaviours during this critical phase [8, 43].

The included studies revealed a pattern of varied adaptations to the photovoice methodology, with differences in focus on action and in implementation. Similar patterns were noted in other photovoice reviews from non-MENA regions, involving both young people [13, 14] and adults [41, 48]**.** While Wang and Burris developed the photovoice process with a set of guiding steps and principles, the modifications observed and the broad range of applications in the reviewed studies reflect not only contextual factors—such as cultural, political, and logistical conditions—but also the method’s theoretical foundations. Because of its theoretical underpinnings, photovoice can be situated within different qualitative methodologies, such as phenomenology, grounded theory, and critical theory, which may help explain the diverse ways it has been applied across the literature [49, 50]. In addition, the participatory and action-oriented nature of photovoice supports this variation, as participatory research itself did not originate from a single discipline. Instead, it has evolved over time into a complex and context-specific approach [51]. This is both a strength and a challenge [51], as it means "there is no fixed formula for designing, practicing, and implementing PAR projects" [52]—including those using photovoice.

In our review, few studies explicitly discussed the reasons behind modifying their photovoice use or elaborated on the theoretical foundations guiding their approach. While logistical factors such as time constraints and budget limitations were mentioned [37], the design and goals of the research also appeared to play a significant role in these adaptations. Although such adjustments might be necessary to meet the needs of the researchers and the population, altering the photovoice methodology in ways that depart from its underlying principles can affect participants’ capacity to advocate for change within their communities [13, 48], and risk tokenizing young people’s participation.

In this review, the primary adaptation was that participants were mostly involved in the photo-taking phase, with limited engagement in other phases. This finding aligns with a recent review of reviews that examined studies predominantly involving adult populations [41] as well as with Yang et al.'s systematic review of photovoice with youth [14]. The latter attributed the limited participation of young people in part to the unique challenges they face, such as “competing demands among school attendance, personal health issues, and family responsibilities” [14]. Additionally, some scholars argue that full participation may not be ideal when working with young people, as it can lead to boredom or overwhelm—particularly for those already navigating complex life circumstances [51, 53]. Moreover, the guiding processes and principles of photovoice or any participatory research were not originally designed with young people in mind [51, 53]. It is therefore preferable to negotiate levels of participation from the outset or when obstacles arise, allowing participants to determine the extent of their involvement [51, 53]. This approach encourages agency and supports the goal of fostering meaningful engagement and respect.

Socio-political factors also contributed to variations in photovoice implementation in a few studies. In low-democratic socio-political environments, restrictions on discussing sensitive topics can further limit young people's engagement. These are not just contextual constraints but reflect deeper structural power dynamics that shape what can be expressed, by whom, and in what settings. For instance, Hayik's research [32, 33] demonstrated challenges during the dissemination phase, which is crucial for achieving the final goal of reaching policymakers. Among Palestinian minority youth in Israel, fears of potential repercussions—such as losing job opportunities—discouraged them from critiquing authorities or sharing their perspectives with wider audiences. These findings suggest that, beyond logistical factors, political and societal pressures may significantly hinder the full implementation of photovoice, particularly in contexts where public critique carries risks.

In addition to these external socio-political constraints, internal power dynamics within photovoice projects—especially between researchers and participants—also play a crucial role in shaping both process and outcomes. However, the limited reporting of reflexivity in the included studies makes it challenging to examine this influence in depth. This limitation may be due to word constraints or the diverse nature of grey literature, such as reports from NGOs that vary widely in purpose, format and focus. Despite these challenges, the photovoice processes reported in the included studies could offer valuable insights into how power dynamics might have influenced the photovoice projects. Most of the projects were externally initiated, often driven by agendas defined by organizations or researchers. While external initiation is not inherently problematic, few studies mentioned efforts to involve young participants in shaping the agenda or defining the topics to be explored [30, 34]. This raises concerns about whether the issues addressed genuinely reflected young people's priorities. Ensuring that photovoice projects align with participants' lived experiences is crucial for fostering meaningful engagement and obtaining authentic responses [15, 16]. Without such involvement, participants may feel pressured to meet researchers' expectations, potentially compromising the credibility of the results. Allowing participants to help define the topics fosters greater engagement and ensures the outcomes are relevant to the young people themselves.

Another aspect of power dynamics that may influence the photovoice process is the limited involvement of young people in analysing or interpreting research findings. Only a few studies engaged participants in this process [30, 34], and this lack of involvement may result in the inadequate representation of young people’s voices. When researchers predominantly conduct the analysis, their perspectives are likely to powerfully shape the findings and conclusions, potentially marginalising young people's own portrayals of their experiences. Due to time constraints, researchers might choose to undertake the analysis themselves but should still look to involve the participants to ensure that knowledge of the particular culture and circumstances is sufficiently reflected in the interpretation and accurately represents what the participants meant.

Nevertheless, although many of the reviewed projects adapted the photovoice methodology in different ways and may have been affected by possible power imbalances influencing the process, they still produced valuable outcomes. Several studies reported that participation in these projects raised awareness and resulted in greater feelings of empowerment among some participants, which may have encouraged personal or social action. This may partly stem from young people’s strong desire to speak and be heard, as they will always find ways to make themselves seen or heard even when adults do not fully provide them the opportunity.

Our review identified five key benefits of using photovoice with young people in the MENA region. While the evidence is limited due to the varying quality of the included studies, these benefits are consistent with those reported in other reviews [13, 48]. Although few studies discussed challenges, those that did were primarily from projects involving minority groups. A significant challenge identified was photovoice’s potential to inadvertently reinforce feelings of marginalisation, due to its powerful influence on project outcomes—both positive and negative [26, 54]. This highlights the importance of strengthened ethical board review and ongoing critical reflexivity among researchers. Ethical engagement in photovoice, or in any participatory research, should be viewed not as a one-time requirement, but as an ongoing process of navigating ethical complexities [55], ensuring meaningful participation, and implementing effective safeguarding throughout the project.

## Strengths and limitations

This is the first scoping review of the literature on photovoice and young people health inequalities in the MENA region. This review followed the JBI and PRISMA-ScR statement guidelines. Multiple bibliographic and organizational databases were searched along with grey literature from google searchers. Literature in English as well Arabic was included.

The scoping review has several limitations. First, young people may have been defined differently from the age criteria we used. We excluded studies that included children under 10 years old or individuals over 24 years old and did not present their findings by age group. Second, the MENA region is linguistically diverse, with French and Farsi as official languages in some countries. Studies published in these languages might have been missed. Additionally, some country-specific journals are not open access and may have contained relevant reports. Finally, these findings were extracted from studies in Egypt, Jordan, Lebanon, Palestine and Saudi Arabia which may not be generalisable to represent the views or experience of the young people in the MENA region.

## Conclusion

Our scoping review highlighted a scarcity of literature on the use of photovoice among young people to address health inequalities and the factors influencing them in the MENA region. Most of this literature comes from non-profit organisation, with few being from peer-reviewed articles. The included studies focused on socio-economically disadvantaged, vulnerable, and marginalised young people, covering topics such as environment, social integration, safety, and youth empowerment. Photovoice implementation varied across studies. There was limited involvement of young people throughout the research process. Most study outcomes went beyond merely raising awareness of the investigated issues to drive tangible, action-oriented change at both personal and immediate community levels, rather than broader societal levels. Key benefits included enhanced communication and expression, as well as increased awareness about the investigated topics, while in the few studies that reported challenges, a primary concern was the fear of negative consequences associated with critiquing or sharing their critique with a wider audience.

### Implications of the findings for future research

Based on the findings of this study, several potential avenues for future research can be proposed. Future research with young people in the MENA region should explore strategies to enhance participant engagement across all phases of the photovoice process, including planning, data analysis, and dissemination. Actively involving young people in these stages will enhance the potential for research outcomes to genuinely reflect their priorities and lived experiences. In some MENA contexts, socio-political dynamics and gender norms may restrict young people’s freedom to participate openly. Research should therefore consider approaches that navigate these dynamics sensitively, such as by ensuring safe spaces for expression and gender-sensitive facilitation. Additionally, further investigation is needed into the ethical challenges associated with photovoice projects, particularly those involving vulnerable groups. Developing frameworks for ethical reflexivity can support researchers in navigating these challenges effectively.

Future studies should also examine how cultural and socio-political factors influence the implementation and outcomes of photovoice projects. Understanding these dynamics can enhance cultural relevance, address power imbalances, improve policy impact, and promote ethically sound methodologies.

Moreover, conducting longitudinal studies can provide valuable insights into the long-term impact of photovoice projects on participants and their communities. This would help assess the sustainability and effectiveness of photovoice as a participatory research and action-oriented method. Finally, future research may employ digital methodologies, including social media and online platforms, to conduct photovoice studies. Exploring these digital approaches can help assess how online engagement expands accessibility, participation, and impact in photovoice initiatives.

## Supplementary Information

Supplementary Material 1.

Supplementary Material 2.

Supplementary Material 3.

Supplementary Material 4.

## Data Availability

No datasets were generated or analysed during the current study.
