# Supplementary material for: Photovoice and health inequalities among young people in the MENA region: Scoping review
Source: Int J Equity Health. 2025 Jun 16;24:176. doi: 10.1186/s12939-025-02527-x (PMC12168262; doi:10.1186/s12939-025-02527-x)
Supplement: Supplementary file 1 — Supplementary Material 1. [file 12939_2025_2527_MOESM1_ESM.pdf]

Peer-reviewed research articles (n=3)						
**#**	**Author(s), Year, document type**	**Country**	**Health related topic covered**	**Aim**	**Photovoice Design**	**Key findings and Outputs**
**1**	**Almughamisi et al (2022a, 2021b)**	Saudi Arabia	Adolescents Childhood obesity	To obtain, through participatory approach, the perspectives of female students, school staff, Ministry of Education representatives and parents on important and feasible intervention opportunities for school-based obesity prevention for adolescent girls.	Photovoice was utilised as part of a mixed-method concept mapping study involving both adult and adolescent stakeholders. It served as a participatory data collection tool to facilitate the generation of statements necessary for the concept mapping study. The study involved 15 adolescents aged 13 to 15 years, and participatory analysis of concept mapping was employed.	The students generated 42 statements from 39 pictures. They identified five themes that had influenced their dietary and physical activity habits: 'Role of Government,' 'School Environment,' 'Home Environment,' 'Retail Environment,' and 'Cultural Practices.'<br><br>Both stakeholder groups agreed on improving access to healthy foods in the canteen.
**2**	**R. Hayik (2021)**	Palestine	Environment	To use Photovoice to document and report problems in the students' environment to an invited audience of influential figures from their community.	Photovoice was framed as a youth participatory action research project and was integrated into English lessons. The study involved 62 students from 10th to 12th grade in high school (adolescents).<br><br>Students were not engaged in the analysis.	The highlighted environmental problems included garbage disposal, environmental pollution, problematic use of public space, and deforestation.<br>74% of students reported that the project had a positive impact on their English learning skills. Writing about self-chosen, relevant topics made English writing more engaging and enjoyable for them. Presentation opportunities enhanced their public speaking skills and boosted their self-confidence.<br>All 62 students stated that their awareness of environmental issues was raised through their engagement, exposing them to previously unknown problems.<br>Students experienced strong emotions such as infuriation, shock, and disappointment, as indicated by their responses. They expressed a willingness to participate in more awareness-raising activities.<br>The students shared their findings at the school with influential community figures such as decision makers, journalists, politicians, and environmentalists, and also at a national professional development conference for English teachers.<br>After the project ended, students became more environmentally active at the local level (e.g., organizing weekly village clean-ups and securing support from the local council for supplies and disposal). Tangible improvements were observed after interacting with the local council (e.g., replacement of broken garbage bins in the village).
**3**	**R. Hayik (2018)**	Palestine	General problems in minority communities.	to raise students' awareness of problematic issues in their surroundings and enable them to highlight these to the wider community.	Photovoice was used as a project in a writing workshop within the English writing course as part of the syllabus. The study involved 29 participants, who were third-year college students (young people).<br><br>Students were not engaged in the analysis.	Students highlighted the following issues: littering, poor infrastructure (n=6), violence (n=5), bad public manners, unhealthy eating habits, child smoking (n=1), and acceptability. They shared their findings in a walk-in photo exhibition with their college professors.<br>The project provided students with new experiences, prompting them to integrate photography into their schoolwork and address societal issues beyond the classroom. Students found the experience eye-opening, raising their awareness of previously overlooked issues. They also gained confidence through the process, particularly in presenting their work. The project encouraged students to brainstorm solutions to these issues.<br>The author noted students' emotional and psychological challenges when addressing sensitive and difficult issues, including fear of retribution and concerns about potential negative impacts on their life chances when tackling socio-political and legally sensitive topics

Grey literature documents (n=8)						
#	Author(s), Year, document type	Country	Health related topic covered	Aim	Photovoice Design	Key findings and Outputs
1	**Peace of Art Organisation (2023)** *News (Eng.)*	Lebanon	Youth empowerment	To empower 30 young people from the North Beka'a area through a comprehensive and intensive series of activities, and life skills development, in parallel with youth leadership, accountability, human rights, advocacy, citizenship, partnerships and networking, and conflict resolution.	-	A town-hall meeting consultation session among stakeholders and activists from the region to address what youth highlighted.
2	**Routes Platform (2022)** *News (Eng.)*	Egypt	Entrepreneurship and improving work opportunities for youth	To use Photovoice and other methods to provide youth with opportunities to access the complex entrepreneurship ecosystem, while focusing on entrepreneurship, impact & sustainability, gender mainstreaming, diversity & inclusion, & wellbeing.	-	-
3	**Wataneya Organisation (2022)** *News (Ar.)*	Egypt	Transition of orphanage to the stage of independence.	To identify the challenges of orphaned youth inside care homes, after they leave the home, and during their transition to the stage of independence.	-	Photo exhibition with messages discussing their challenges, dreams, and stories inside and outside care homes. Two discussion sessions with the youth and decision-makers from various fields, focusing on the role of youth in community development and the role of active entities in integrating and supporting youth during their journey to independence.
4	**Naba'a and Family for Every Child (2022)** *Non-profit organisation report*	Lebanon	Integration of adolescent's refugees	To identify barriers and challenges of integration of children on the move in Lebanon, providing recommendations for improvement, and supporting the development of practical guidelines.	The photovoice method was part of a multi-method study utilized in this project. It involved participatory research with six adolescent refugees aged 15-20 years. Used participatory analysis	The adolescents reported their findings of experiencing a contradiction between their rights and actual experiences. They felt leisure activities were inaccessible, believed integration into Lebanon was a waste of time, and viewed European countries as better prospects for integration.<br><br>The exhibition enabled young refugees, who felt invisible, to have their voices heard.
5	**R. Hayik (2020)** *Book chapter*	Palestine	Linguistic landscape and language diversity	To use photovoice to raise students' awareness of linguistic landscape issues, encourage them to advocate for fair visibility of their minority language.	Photovoice was utilized as a homework assignment in the English writing course as part of the syllabus. The study was conducted with 20 young people aged 18 to 21 years.<br><br>Students were not engaged in the analysis.	The students photovoice addressed several issues: the absence of Arabic from many signs (n= 10), the abundance of mistakes in Arabic words (n=6), and the Hebraization of many Arabic names of locations (n=4). The students shared their findings with the local community.<br>All students mentioned that their participation in the Photovoice process increased their awareness of local issues. They explained how the experience heightened their attentiveness to their surroundings and expressed surprise at the realities they uncovered.<br>The author reported that the awareness raised through Photovoice had a strong negative emotional impact on students, leaving them feeling marginalized. They also faced emotional and psychological challenges when addressing sensitive and difficult issues.<br>Students chose not to present their work to an influential audience due to concerns about jeopardizing their future employment prospects in the education sector, where they aspire to become teachers

Grey literature documents (n=8)						
#	Author(s), Year, document type	Country	Health related topic covered	Aim	Photovoice Design	Key findings and Outputs
6	**Malherbe et al, (2018)**<br><br>*Book chapter*	Egypt	Safety during times of conflict.	To illustrate the ways in which Photovoice initiatives allow young people to speak to, disrupt as well as highlight, their experiences and depictions of socio-political transition.	A multi-country project invited young people to photograph things, places, and people that made them feel safe or unsafe in their communities, aiming to highlight youth perspectives on safety within marginalized communities.<br><br>This book chapter focuses on the Egyptian component, which explored the experiences of young Egyptians during the country's socio-political transition. The study was based on 26 adolescents aged 12 to 16 years and included researcher-led analyses.<br><br>Students were not engaged in the analysis due to time, resources and budget constraints.	The photo-stories revealed how safety among Egyptian youth was related to police presence, economic security, improved quality of life, and communities caring for each other.<br><br>Following the first phase of project implementation, three youth-centric discursive spaces were co-created by the participants and the local research team to extend the project's agenda of raising social consciousness beyond the photo-stories.
7	**PhotoVoice Organisation (2014)**<br><br>*Non-profit organisation report*	Jordan	Displacement due to sexual violence	to capture and share young Syrian refugees lived experiences, particularly concerning their displacement due to sexual violence and share realities and struggles to the world.	PhotoVoice partnered with World Vision UK on a project involving Syrian refugee and host community youth in Jordan. The project engaged two distinct groups of young men and women, aged 14 to 20, who provided valuable insights into their own experiences and the resulting impacts.	Young refugee individuals experienced a range of emotions, including sadness, disorientation, anger, and confusion, as they perceived injustice towards Syria and felt there was an inadequate global response. Despite these challenges, they maintained optimism about the future. They expressed faith in the potential of political leaders to effect change and in their own voices as agents of transformation, believing this could eventually facilitate their return home.<br><br>The participants approached the photovoice project with enthusiasm, creatively combining visual and written language to express their viewpoints.<br><br>Their work was showcased at a local celebration with over 150 attendees and at the Global Summit to End Sexual Violence in Conflict in London. World Vision integrated photography into the curriculum for their child and adolescent friendly space programs at two community centres in Jordan.
8	**PhotoVoice Organisation (2008)**<br><br>*Non-profit organisation report*	Palestine-Israel	Conflict in Palestine and Israel.	to create dialogue and mutual understanding as a means to end the conflict in Palestine and Israel.	PhotoVoice collaborated with an NGO in Jerusalem to foster dialogue and mutual understanding as a means of addressing the conflict between Palestine and Israel. The Side-by-Side project united six Palestinian teenagers and six Israeli teenagers, aged 13-18, from across Israel and the West Bank. All participants were members of the Families Forum, a Palestinian-Israeli organization comprising bereaved families dedicated to non-violence and dialogue.	The participants' images facilitated important dialogue among adolescents on both sides of the Arab Israeli conflict, enabling them to voice their losses, frustrations, and differences.<br><br>Participants shared their work on a project-specific interactive website, where they could view and comment on each other's contributions. The images fostered significant dialogue among teenagers on both sides. Exhibitions of the youths' work to Israeli, Palestinian, and international audiences heightened awareness of dialogue and cooperation
