# Supplementary material for: Photovoice and health inequalities among young people in the MENA region: Scoping review
Source: Int J Equity Health. 2025 Jun 16;24:176. doi: 10.1186/s12939-025-02527-x (PMC12168262; doi:10.1186/s12939-025-02527-x)
Supplement: Supplementary file 2 — Supplementary Material 2. [file 12939_2025_2527_MOESM2_ESM.pdf]

Table 4. Quality Assessment of Peer-reviewed Research Articles			
**the JBI qualitative checklist**	**Almughamisi et al (2022)**	**Hayik (2021)**	**Hayik (2018)**
**Is there congruity between the stated philosophical perspective and the research methodology?**	Yes	Yes	Yes
**Is there congruity between the research methodology and the research question or objectives?**	Yes	Yes	Yes
**Is there congruity between the research methodology and the methods used to collect data?**	Yes	No	No
**Is there congruity between the research methodology and the representation and analysis of data?**	Yes	No	No
**Is there congruity between the research methodology and the interpretation of results?**	Yes	No	No
**Is there a statement locating the researcher culturally or theoretically?**	Unclear	Yes	Yes
**Is the influence of the researcher on the research, and vice- versa, addressed?**	Unclear	Unclear	Unclear
**Are participants, and their voices, adequately represented?**	N/A	Yes	Yes
**Is the research ethical according to current criteria or, for recent studies, and is there evidence of ethical approval by an appropriate body?**	Yes	Unclear	Unclear
**Do the conclusions drawn in the research report flow from the analysis, or interpretation, of the data?**	Yes	Yes	Yes
**Evaluation method used**	N/A	n=62 (All participants) students' reflections after the end of the process. Content analysis was conducted	n=29 (All participants) students' reflections through a focus group interview after the end of the process. Content analysis was conducted
**Overall appraisal**	Include	Include	Include

Table 5 Method of Photovoice and Ethical considerations (Peer-reviewed research articles)

#	Author(s), Year	Rationale of use	Photovoice methodology	Ethical Aspects and Consideration
**1**	**Almughamisi et al (2022a, 2021b)**	Used for effective engagement of adolescents and the generation of meaningful conceptualisation of the issues being investigated.	Purposeful recruitment included 6 administrative staff, 2 canteen staff, and 6 Ministry of Education staff responsible for nutrition and health in the participating schools. Two intermediate schools were purposively identified, and 15 students were randomly recruited from each year of each school.<br><br>An introductory session was held to explain the project goals and objectives. Students were instructed to take photos with their phone cameras for one week and email them to the researcher.<br>Research prompts were collaboratively developed.<br><br>Group discussions of photographs utilized the SHOWeD technique to generate statements, and participatory analysis involved concept mapping.	Ethical approvals were received from both national and international institutional review boards.<br><br>Written informed consent was obtained from the individuals and, in the case of minors, from their legal guardians or next of kin prior to all student activities.
**2**	**R. Hayik (2021)**	Used to engage marginalized ethnic groups in the environmental public discourse and for its the capacity as an innovative tool to raise awareness about issues and potential solutions.	The researcher collaborated with three English teachers and one environmental studies teacher at a participating school to implement Photovoice in classrooms (grades 10-12). Training involved English teachers familiarizing themselves with Photovoice through academic articles and professional development in writing-teaching methodology. The environmental studies teacher conducted workshops on environmental challenges to enhance students' awareness of potential issues to explore.<br><br>Photovoice was integrated into English lessons, offering students the option to complete the assignment as part of their learning tasks. Researchers provided prompts, and each student took one photo.<br>Students individually wrote descriptions of their photos, explaining their choices and suggesting improvements. English teachers supported students throughout the writing process and held individual conferences to help students strengthen their writing. Researchers conducted a formal analysis, including content analysis of students' Photovoice projects and reflections collected at the project's conclusion.	No ethical approval from the ethics committee was mentioned.<br><br>Signed informed consents were obtained from students who agreed to participate in this study by the end of the project.
**4**	**R. Hayik (2018)**	Used to create possibilities for marginalised young people to become socially and politically engaged.<br><br>A creative, empowering, non-violent tool for expressing protest while improving literacy skills and nurturing more confident students who are willing to speak up	The photovoice study was part of the syllabus for the English Methodology course for English as a Foreign Language, focusing on how students could make their voices heard through writing and other forms of expressive communication.<br>Students familiarized themselves with photovoice by reading articles and engaging in discussions, including considerations of the ethics of photography. They received prompts from the researcher but had the freedom to choose their subjects and how they would write about them.<br>Students used their photos in a writing workshop, applying principles of process writing. They received support and feedback from their English teacher and peers, focusing exclusively on writing skills. There was no discussion of the issues they chose to depict; the focus was solely on revising their writing.<br>The researcher conducted a content analysis of the students' photovoice projects and the transcripts of the students' focus group reflections. Students did not participate in the analysis.	No ethical approval from the ethics committee was mentioned.<br><br>Signed release consents and approval to use the pictures for future publication were obtained from the people whom the students photographed.<br><br>The researcher addressed students' concerns about public sharing and potential risks to themselves by ensuring a safe space where they could share with trusted individuals.

Table 5 Method of Photovoice and Ethical considerations (non-peer-reviewed documents)

#	Author(s), Year	Rationale of use	Photovoice methodology	Ethical Aspects and Consideration
**1**	**Naba'a & Family for Every Child (2022)**<br><br>*NPO Report*	Used to empower marginalised participants, provides a safe and creative space to use photography for addressing difficult topics and concerns, and advocacy, stimulates interest in important community issues and enable communication of diverse groups through the art of photography.	The project was initiated with support from Family for Every Child global alliance. Participants received basic training in photography, visual literacy, storytelling, and the ethics of photography as part of the research project background. The process involved six workshops, with fieldwork conducted over two weeks. Researchers provided prompts, and participants had half a day to take photos. Group discussions and participatory analysis followed, with participants identifying common themes across the images. Participants, in collaboration with facilitators, decided on the target audience and the messages they wished to convey. From the 250 photographs taken, they selected 50 images that they believed best represented these messages.	An ethical protocol was developed by Family for Every Child and Naba'a. Consent was obtained from research participants through signed consent forms or oral consent, ensuring their agreement to participate.<br><br>Naba'a implemented its Child Safeguarding Policy. The detailed ethics procedures are covered in a separate report.
**2**	**R. Hayik (2020)**<br><br>*Book chapter*	Used to engage students in a meaningful writing experience, offering empowering alternative to traditional writing exercises and raise awareness of community issues.	The Photovoice project was integrated into the syllabus of an English writing course for EFL learners as a homework assignment, focusing on addressing linguistic landscape issues in their community and assessing their writing skills progression through multiple drafts.<br>Students familiarized themselves with Photovoice by reading and discussing articles.<br>The researcher provided a prompt allowing students the freedom to choose and photograph as many pictures as they wanted over one week, to be discussed in the next session. A list of guiding questions was given to examine linguistic landscapes, including critical inquiries, personal insights, and suggestions for change. Responsive individual feedback and mini sessions were provided to help students improve their writing. The researcher conducted a content analysis of the students' photovoice projects and the transcripts of focus group student reflections. Students did not participate in the analysis.	No ethical approval from the ethics committee or consent activities were mentioned.<br><br>The researcher acknowledged participants' fear of criticizing authorities and chose not to present to an invited audience, respecting their need to protect their future employment prospects.
**3**	**Malherbe et al (2018)**<br><br>*Book chapter*	Photovoice was utilized as a socially conscious tool, providing participants with a creative platform to navigate and comprehend complex socio-political discourses and empower participants by facilitating critical dialogue with adults and fostering their leadership in social justice campaigns.	The project was part of a multi-country Photovoice initiative initiated by the University of South Africa.<br>The authors did not specify whether these discussions were individual or group dialogues. Researchers conducted the analysis, and students did not participate in the analysis due to time constraints.	–
**4**	**Photovoice (2014)**<br><br>*NPO Report*	–	PhotoVoice partnered with World Vision UK on a project involving Syrian refugee and host community youth in Jordan, working with two separate groups of young men and women.	–
**5**	**PhotoVoice (2008) Palestine – Israeli**<br><br>*NPO Report*	–	PhotoVoice organization collaborated with a local NGO. The Side-by-Side project was launched during a three-day workshop.<br>Over the next eight months, aided by local facilitators, young people documented their lives using digital cameras. Participants convened four times at photographic and dialogue camps held in the West Bank and Israel. Local facilitators visited young people in their respective areas to offer additional guidance and support. Between workshops, participants had access to computers and digital cameras.	–
