# Supplementary material for: Photovoice and health inequalities among young people in the MENA region: Scoping review
Source: Int J Equity Health. 2025 Jun 16;24:176. doi: 10.1186/s12939-025-02527-x (PMC12168262; doi:10.1186/s12939-025-02527-x)
Supplement: Supplementary file 3 — Supplementary Material 3. [file 12939_2025_2527_MOESM3_ESM.pdf]

MEDLINE Search Strategy

1. (photovoice or "photo voice" or "participa* photo*" or "photo novella" or photonovella or "photo stor*" or "reflex* photo*" or "photo* interview*").mp. [mp=title, book title, abstract, original title, name of substance word, subject heading word, floating sub-heading word, keyword heading word, organism supplementary concept word, protocol supplementary concept word, rare disease supplementary concept word, unique identifier, synonyms, population supplementary concept word, anatomy supplementary concept word]
2. (participation adj3 research).mp.
3. exp Community-Based Participatory Research/
4. 2 or 3
5. exp Photography/
6. photo*.tw.
7. 5 or 6
8. 4 and 7
9. 1 or 8
10. africa, northern/ or algeria/ or egypt/ or libya/ or morocco/ or tunisia/ or sudan/ or middle east/ or bahrain/ or iraq/ or jordan/ or kuwait/ or lebanon/ or oman/ or qatar/ or saudi arabia/ or syria/ or united arab emirates/ or yemen/ or djibouti/ or arabs/ or iran/ or israel/ or (algeria* or bahrain* or egypt* or iraq* or jordan* or kuwait* or kuweit* or leban* or liban* or lubnan* or lobnan* or yemen* or aden or sanaa or UAE or Emirat* or (abu adj dhabi) or dubai or libya* or morocco or moroccan* or oman* or muscat or palestin* or ghazza or ghaza or gaza or (west* adj2 bank) or q?atar* or katar* or saudi* or KSA or Syria* or tunis* or ((east* or north*) adj2 africa*) or sudan* or djibouti* or iran* or israel* or ifni or (trucial adj state*) or MENA or EMRO or ((middle or near) adj2 east*) or (east* adj2 mediterranean) or orient or arabs or arab or arabia or levant).mp.
11. exp Africa, Northern/
12. exp middle east/
13. 10 or 11 or 12
14. 9 and 13

Google Search (Arabic Keywords)

**Find all these words:**	فوتوفويس OR photovoice
**Any of these words:**	الجزائر OR البحرين OR جيبوتي OR مصر ORإيران OR العراق OR الأردن OR الكويت OR لبنان OR ليبيا OR المغرب OR عُمان OR قطر OR المملكة العربية السعودية OR دولة فلسطين OR السودان OR الجمهورية العربية السورية OR تونس OR الإمارات العربية المتحدة OR اليمن
**Language:**	Arabic

Google Search (English Keywords)

**Find all these words:**	Photovoice
**Any of these words:**	Algeria OR Bahrain OR Djibouti OR Egypt OR Iran OR Iraq OR Jordan OR Kuwait OR Lebanon OR Libya OR Morocco OR Oman OR Qatar OR Saudi Arabia OR State of Palestine OR Sudan OR Syrian Arab Republic OR Tunisia OR United Arab Emirates OR Yemen
**Language:**	English
